# Inequity in Access to and Use of Digital Health Technologies in Routine Heart Failure Care: Protocol for a Scoping Review

**DOI:** 10.2196/81949

**Published:** 2026-01-23

**Authors:** Nicklas Vinter, Mariam Elmegaard, Lars Køber, Morten Schou, Søren Paaske Johnsen

**Affiliations:** 1 Danish Center for Health Services Research, Department of Clinical Medicine, Aalborg University, Selma Lagerløfs Vej 249, Aalborg, 9260, Denmark, 45 25321675; 2 Department of Cardiology, Regional Hospital Central Jutland, Viborg, Denmark; 3 Department of Cardiology, Herlev and Gentofte University Hospital, Copenhagen, Denmark; 4 Department of Cardiology, Rigshospitalet, Copenhagen University Hospital, Copenhagen, Denmark

**Keywords:** heart failure, digital health technology, routine practice, inequity, review

## Abstract

**Background:**

Heart failure (HF) is a global health challenge characterized by high mortality, morbidity, and economic burden. The development of digital health technologies offers promising tools for prevention, early detection, and management of HF, potentially improving prognoses and reducing costs. However, these innovations may also widen existing health disparities related to socioeconomic status, geography, and race/ethnicity.

**Objective:**

This scoping review will examine and map existing evidence on socioeconomic, geographic, and racial/ethnic differences in access to and use of digital health technologies for HF care in routine practice.

**Methods:**

The writing of this protocol followed the methodological framework by Arksey and O’Malley, including (1) identifying the research question; (2) identifying relevant studies; (3) selecting studies to be included in the review; (4) charting the data; and (5) collating, summarizing, and reporting the results. Eligible studies must examine digital health technologies in adults (aged ≥18 years) with any type of HF and report on social determinants of health, geography, or race/ethnicity. Observational study designs will be included. Searches will be conducted in Embase, PubMed, Google Scholar, and Scopus. A 2-stage screening process will determine study eligibility, and data will be extracted using a standardized form.

**Results:**

The project is funded. Data collection is expected to begin by the beginning of 2026.

**Conclusions:**

This scoping review will map existing evidence on differences in access to and use of digital health technologies for HF care. The findings are anticipated to highlight patterns and gaps in the literature, informing future research and strategies for equitable implementation.

## Introduction

Heart failure (HF) is a complex clinical syndrome that represents a considerable global health burden, characterized by high mortality, morbidity, and costs [1,2]. The development of digital health technologies for HF is expanding, supporting primary prevention, early detection, and disease management [3,4], and recent reviews highlight the growing role of remote monitoring, wearable technologies, and integrated care models [5-8]. Application of digital health tools in health care services and decision-making has the potential to improve patient care and clinical outcomes and reduce health care expenditures. However, comprehensive investigations are needed to understand the broader implications, including ethical, practical, and systemic concerns, associated with implementation.

Understanding the consequences for health equity of implementing digital health technologies is essential [9]. Health inequities based on socioeconomic status, race/ethnicity, and geography remain a significant challenge in HF care. Inequities can lead to unequal health outcomes, with certain groups experiencing higher rates of hospitalization, poorer quality of life, and increased risk of adverse clinical outcomes [10-14]. While digital health innovations may offer new opportunities for care delivery, uneven access and use across population groups could exacerbate existing inequities. Recent work emphasizes the need for equity frameworks in digital health implementation [15].

Without deliberate efforts to ensure equitable implementation, the digital transformation of health care risks reinforcing or even widening inequities [16,17], yet the extent and nature of evidence available in the literature regarding patients with HF remain unclear. To address this gap, we will conduct a scoping review of digital health technologies used in HF care. The objective of this scoping review is to map and synthesize what is known from existing literature about socioeconomic, geographic, and racial/ethnic differences in access to and use of digital technologies in routine clinical practice among individuals with HF. We hypothesize that individuals from underserved socioeconomic groups, rural or remote areas, or racial/ethnic minority groups have lower access to and use of digital health technologies.

## Methods

### Overview

**Figure 1.**
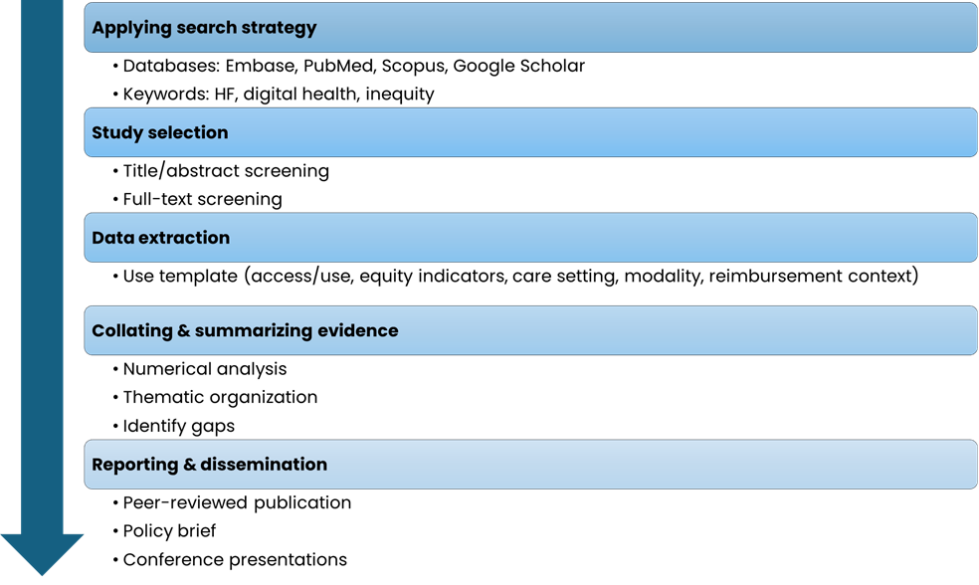
Overview of the scoping review workflow. HF: heart failure.

The approach follows the methodological framework by Arksey and O’Malley [18] and incorporates refinements proposed by Levac et al [19] and guidance from the Joanna Briggs Institute (JBI) for scoping reviews to enhance methodological rigor [20]. We will adhere to the PRISMA-ScR (Preferred Reporting Items for Systematic reviews and Meta-Analyses extension for Scoping Reviews) checklist when reporting the review [21]. This protocol was preregistered at Open Science Framework [22]. Figure 1 illustrates the overall workflow of the scoping review.

### Eligibility Criteria

Textbox 1 summarizes the inclusion and exclusion criteria. We will include studies on individuals aged ≥18 years with any type of HF that report on any digital health technology. We will use the World Health Organization (WHO) definition of “digital health intervention,” which states that such an intervention involves the use of digital technology to achieve specific health-related objectives, and that it is integrated into digital health applications and information and communication technology systems [9]. Data on social determinants of health, geography, and race/ethnicity must also be present in the studies. The selection of the social determinants of health was inspired by previous studies on HF (Table 1) [23]. Any study with an observational design is eligible, because this design reflects real-world access and use patterns in routine practice. Randomized controlled trials and qualitative-only studies are excluded as they often occur in controlled settings that may mask real-world inequities. Eligible studies must provide data on access to or use of the digital health technology. We will place no restriction on the calendar year of publication. Finally, we will include only full-text articles published in English in peer-reviewed journals. The restriction is due to resource limitations for translation and to ensure methodological rigor. We acknowledge that this may introduce language and publication bias, which will be discussed as a limitation.

Textbox 1. **Inclusion and exclusion criteria.**

**Inclusion criteria**

- Adults (≥18 years) with any type of heart failure
- Studies reporting on digital health technologies (World Health Organization definition)
- Observational studies (cross-sectional, cohort, case-control)
- Studies that report social determinants of health, geography, or race/ethnicity
- Full-text articles published in English in peer-reviewed journals
- Any publication year

**Exclusion criteria**

- Randomized controlled trials, qualitative-only studies
- Conference abstracts, editorials, letters, non–peer-reviewed sources
- Non–English language publications

**Table 1.** Domains of social drivers used to examine inequity.

Data domain	Definition
Social determinants of health	Insurance Income/wealth Marital status Educational attainment Neighborhood socioeconomic status Social support Employment status Health literacy
Geography	Urban/rural residency Distance to hospital Administrative regions
Race and ethnicity	Any group

### Search Strategy and Information Sources

A professional health care librarian was consulted to build the search strategy (Table 2). Our search strategy combines HF and digital health technology, and then either inequity, social determinants of health, geography, or race/ethnicity. Databases to be searched include Embase, PubMed, and the interdisciplinary sources Google Scholar and Scopus.

**Table 2.** Search strategy. Search query: number 1 AND number 2 AND (number 3 OR number 4 OR number 5 OR number 6).

Number	Topic	Database			
		Embase	PubMed	Google Scholar	Scopus
1	Heart failure	“heart failure”/exp OR “heart failure”	“Heart Failure” [Mesh] OR “heart failure”	“heart failure”	“heart failure”
2	Digital health intervention	(“digital intervention”/exp OR “digital intervention” OR (“digital” AND (“intervention”/exp OR “intervention”))) OR (“digital health”/exp OR “digital health”) OR (“mobile application”/exp) (“information technology device”/exp) OR (“digital technology”/exp) OR (“telehealth”/exp) OR (“mhealth”/exp) OR “mhealth” OR “mobile health”	“Telecommunications” [Mesh] OR “digital intervention” OR “digital health intervention” OR (“digital” AND “intervention”) OR “mHealth” OR “mobile health”	“digital intervention” OR “mHealth” OR “mobile health”	“digital intervention” OR (“digital” AND “intervention”) OR “mHealth” OR “mobile health”
3	Health inequity	“health disparity”/exp OR “disparity in health” OR “health disparities” OR “health economic disparity” OR “health inequality” OR “health inequities” OR “health inequity” OR “health social disparity” OR “health social economic disparity” OR “health social inequality” OR “health socio-economic disparity” OR “health socioeconomic disparity” OR “health socioeconomic inequity” OR “health status disparities” OR “health status disparity” OR “health status inequality” OR “health status inequity” OR “inequality in health” OR “inequity in health” OR “socioeconomic disparities in health” OR “health disparity”	“Health Inequities” [Mesh] OR “inequity” OR “inequities” OR “disparity” OR “disparities”	“inequity” OR “disparity”	“inequity” OR “inequities” OR “disparity” OR “disparities”
4	Social determinants of health	“social determinants of health”/exp OR “social determinant” OR “social determinants” OR “social determining factor” OR “social factors determining health” OR “social health determinant” OR “social determinants of health”	“Social Determinants of Health” [Mesh] OR “social determinant of health”	“Social Determinants of Health”	“Social Determinants of Health” OR “social determinant of health”
5	Geographic variation	“geography”/exp OR “geographic factor” OR “geographic locations” OR “geography” OR “geography”/exp OR “geographic factor” OR “geographic locations” OR “geography” OR “geographic distribution”/exp OR “distribution, geographic” OR “geographical distribution” OR “geographic distribution” OR “geographic variation”/exp OR “urban area”/exp OR “built-up area” OR “built-up land” OR “urban environment” OR “urban land” OR “urbanized area” OR “urbanized environment” OR “urbanized land” OR “urbanized area” OR “urbanized environment” OR “urbanized land” OR “urban area” OR “rural area”/exp OR “agricultural area” OR “rural environment” OR “rural land” OR “rural area”	“Population” [Mesh] OR “geographic variation” OR “geography”	—^a^	“geography” OR “geographic variation” OR “rural” OR “urban”
6	Race/ ethnicity	“ethnic group”/exp OR “ethnic and racial groups” OR “ethnic and racial minorities” OR “ethnic groups” OR “ethnic minorities” OR “ethnic minority” OR “ethnic origin” OR “ethnic population” OR “ethnic status” OR “ethno-linguistic group” OR “ethnolinguistic group” OR “ethnic group”	“Ethnicity” [Mesh] OR “ethnicity” OR “ethnic” OR “Racial Groups”[Mesh] OR “race”	—	“ethnicity” OR “ethnic” OR “race” OR “racial”

a Not applicable.

### Study Selection

Two independent reviewers (NV and ME) will use a 2-step screening approach. First, all identified records will be manually screened for eligibility based on titles and abstracts. Second, the full-text reports of the qualifying abstracts will be assessed for eligibility. Finally, the 2 reviewers will screen the list of references of the selected studies. Any inconsistencies will be resolved by formal consensus, which will involve SPJ.

### Collecting and Charting Data

Based on the identified literature, NV and ME will independently extract data following the data charging form: (1) authors and year of publication (2); study design and population; (3) aims of the study (4); study location and calendar period; (5) care setting (eg, inpatient, outpatient, or home-based); (6) digital health technology modality; (eg, telemonitoring, mobile app, or wearable); (7) operational definitions of “access” (availability and ability to obtain or use technology) and “use” (actual engagement or use); (8) equity indicators (income, education, race/ethnicity, geography, and insurance status); (9) reimbursement and connectivity context (insurance coverage, internet access, and device ownership); (10) primary outcome measure; (11) main findings, reported according to age, sex, socioeconomic characteristics, geographic characteristics, and race/ethnicity.

### Collating, Summarizing, and Reporting the Results

We will systematically organize and present the findings.

#### Numerical Analysis

Reported measures of association (eg, odds ratios, risk ratios, and hazard ratios) will be tabulated and organized by age, sex, social determinants, geography, and race/ethnicity to examine patterns of inequity. Relevant measures of association may be, for example, high-income vs low-income or urban vs rural residency. We will consider differences in health outcomes as health inequities if they are systematic, avoidable, unnecessary, unfair, and unjust [24]. No meta-analysis or pooled effect estimates will be performed; findings will be summarized narratively.

#### Thematic Organization

Data from each identified study will be reported by type of digital intervention, which will follow the WHO classification. The WHO classification includes interventions for patients with HF (referred to as clients by the WHO), interventions for health care providers, interventions for health systems or service managers, and interventions for data services [25]. The relevance of the WHO classification system lies in its ability to link interventions with a list of health system challenges, thereby illustrating how technology may address health care needs. Furthermore, it provides easy access to results for researchers planning new randomized controlled trials. Data charting will follow the iterative process recommended by the JBI. We will first pilot the data charting form on a small sample of included studies to ensure all relevant variables (eg, equity indicators and digital modality) are captured. Next, we will refine the form by adding or adjusting fields as new concepts emerge during extraction.

#### Identification of Research Gaps

We will highlight areas where research is lacking or where findings are inconsistent. Stakeholder consultation will be integrated during interpretation of findings to validate relevance and identify gaps. This will involve engaging clinicians only, who will review preliminary findings and provide input on practical implications.

### Methodological Appraisal

To contextualize our interpretation of inequities, we will include a structured appraisal of key methodological features using a checklist adapted from JBI guidance [20]. The appraisal will be descriptive and will not involve scoring or exclusion. The checklist will include the following domains:

1. Study design
2. Sample size adequacy
3. Completeness of equity-related reporting (eg, socioeconomic status, geography, race/ethnicity)
4. Clarity of definitions for access and use

Findings from this appraisal will be summarized narratively to highlight major limitations and inform interpretation.

## Results

**Figure 2.**
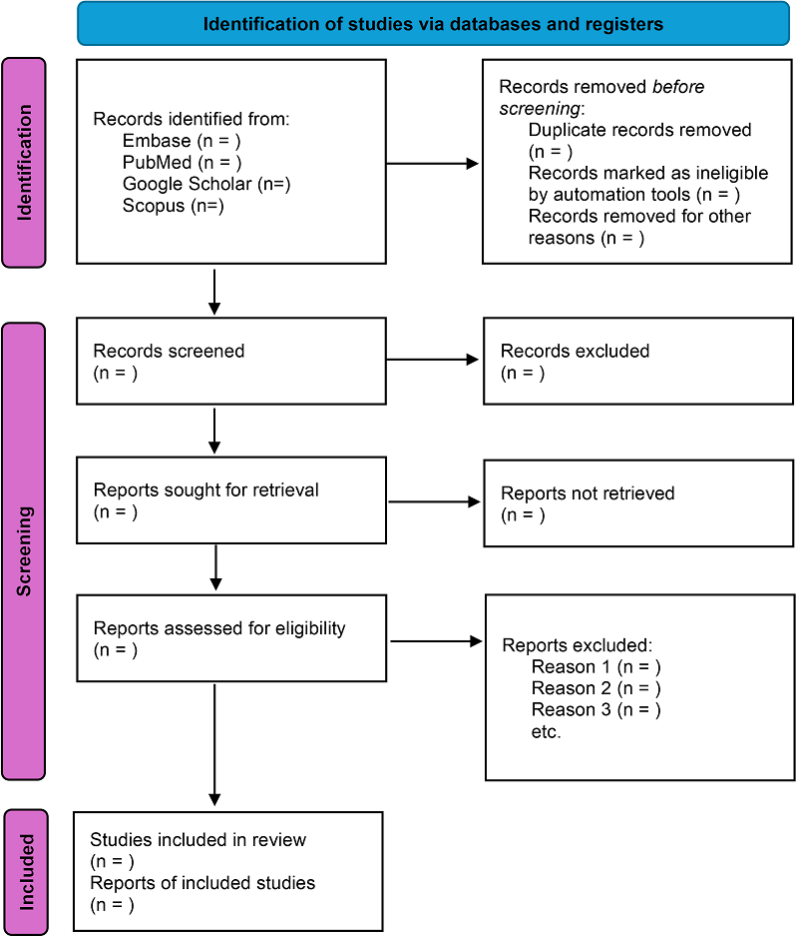
PRISMA (Preferred Reporting Items for Systematic reviews and Meta-Analyses) flowchart.

The project is funded. Data collection is expected to begin by the beginning of 2026. A PRISMA 2020 flow diagram will be included in the final review to illustrate the study selection process. Figure 2 shows a blank version of the flowchart, which will be updated once screening and inclusion are completed.

## Discussion

### Anticipated Main Findings

This scoping review will map existing evidence on socioeconomic, geographic, and racial/ethnic differences in access to and use of digital health technologies for HF care. By synthesizing patterns across observational studies, the review aims to identify where inequities may exist and highlight gaps in the literature. The findings will inform future research and guide strategies for equitable implementation of digital health solutions in routine practice.

### Future Directions

The review will identify patterns and gaps in the evidence base, informing the design of future studies and interventions aimed at reducing inequities in digital health adoption. Findings may also support policymakers and health care organizations in developing strategies to ensure equitable access to digital technologies for HF care.

### Limitations

A key limitation of this review is the restriction to English-language, peer-reviewed full texts, which may introduce language and publication bias. The restriction is particularly relevant for equity-focused reviews, and future research should consider multilingual searches and inclusion of gray literature to minimize bias.

### Dissemination Plan

Results will be disseminated through a peer-reviewed publication, policy briefs, and presentations at conferences and stakeholder meetings, ensuring accessibility to researchers, clinicians, and decision-makers.

### Conclusion

The review will synthesize evidence on disparities in digital health adoption for HF care, offering insights to inform policy, practice, and research agendas aimed at reducing inequities. Findings will highlight gaps in current knowledge and support the design of interventions that promote inclusive digital health strategies.

## Abbreviations

HF: heart failure
JBI: Joanna Briggs Institute
PRISMA-ScR: Preferred Reporting Items for Systematic Reviews and Meta-Analyses extension for Scoping Reviews
WHO: World Health Organization

## References

[1] Bragazzi NL, Zhong W, Shu J (2021). Burden of heart failure and underlying causes in 195 countries and territories from 1990 to 2017. Eur J Prev Cardiol.

[2] Virani SS, Alonso A, Aparicio HJ (2021). Heart disease and stroke statistics-2021 update: a report from the American Heart Association. Circulation.

[3] Cowie MR, McBeath KCC, Angermann CE (2022). The digital future of heart failure care. Curr Heart Fail Rep.

[4] Farwati M, Riaz H, Tang WHW (2021). Digital health applications in heart failure: a critical appraisal of literature. Curr Treat Options Cardiovasc Med.

[5] Azizi Z, Broadwin C, Islam S (2024). Digital health interventions for heart failure management in underserved rural areas of the United States: a systematic review of randomized trials. J Am Heart Assoc.

[6] Noci F, Capodici A, Nuti S, Passino C, Emdin M, Giannoni A (2025). Wearable technologies to predict and prevent and heart failure hospitalizations: a systematic review. Eur Heart J Digit Health.

[7] Kallas D, Sandhu N, Gandilo C (2023). Use of digital health technology in heart failure and diabetes: a scoping review. J Cardiovasc Transl Res.

[8] Zwack CC, Haghani M, Hollings M (2023). The evolution of digital health technologies in cardiovascular disease research. NPJ Digit Med.

[9] (2019). Recommendations on Digital Interventions for Health System Strengthening. World Health Organization.

[10] Lewsey SC, Breathett K (2021). Racial and ethnic disparities in heart failure: current state and future directions. Curr Opin Cardiol.

[11] Lawson CA, Zaccardi F, Squire I (2019). 20-year trends in cause-specific heart failure outcomes by sex, socioeconomic status, and place of diagnosis: a population-based study. Lancet Public Health.

[12] Schjødt I, Johnsen SP, Strömberg A, Kristensen NR, Løgstrup BB (2019). Socioeconomic factors and clinical outcomes among patients with heart failure in a universal health care system. JACC Heart Fail.

[13] Witte KK, Patel PA, Walker AMN (2018). Socioeconomic deprivation and mode-specific outcomes in patients with chronic heart failure. Heart.

[14] Vinter N, Fawzy AM, Gent D (2022). Social determinants of health and cardiovascular outcomes in patients with heart failure. Eur J Clin Invest.

[15] Bitomsky L, Pfitzer EC, Nißen M, Kowatsch T (2024). Advancing health equity and the role of digital health technologies: a scoping review protocol. BMJ Open.

[16] Price-Haywood EG, Arnold C, Harden-Barrios J, Davis T (2023). Stop the divide: facilitators and barriers to uptake of digital health interventions among socially disadvantaged populations. TOJ.

[17] Cheng C, Beauchamp A, Elsworth GR, Osborne RH (2020). Applying the electronic health literacy lens: systematic review of electronic health interventions targeted at socially disadvantaged groups. J Med Internet Res.

[18] Arksey H, O’Malley L (2005). Scoping studies: towards a methodological framework. Int J Soc Res Methodol.

[19] Levac D, Colquhoun H, O’Brien KK (2010). Scoping studies: advancing the methodology. Implement Sci.

[20] Peters MDJ, Marnie C, Tricco AC (2020). Updated methodological guidance for the conduct of scoping reviews. JBI Evid Synth.

[21] Tricco AC, Lillie E, Zarin W (2018). PRISMA Extension for Scoping Reviews (PRISMA-ScR): checklist and explanation. Ann Intern Med.

[22] Digital health technologies in heart failure and health inequity. A scoping review of access and use in routine practice. Open Science Framework.

[23] Enard KR, Coleman AM, Yakubu RA, Butcher BC, Tao D, Hauptman PJ (2023). Influence of social determinants of health on heart failure outcomes: a systematic review. J Am Heart Assoc.

[24] Braveman P (2014). What are health disparities and health equity? We need to be clear. Public Health Rep.

[25] Melh G, Tamrat T (2018). Classification of Digital Health Interventions v1.0. World Health Organization.

